# Measurement of third-order elastic constants and applications to loaded structural materials

**DOI:** 10.1186/s40064-015-1019-2

**Published:** 2015-07-07

**Authors:** Sennosuke Takahashi, Ryohei Motegi

**Affiliations:** National Research Institute for Metals, 6-13-12 Kanamori, Machida, Tokyo, 194-0012 Japan

**Keywords:** Third-order elastic modulus, Elastic wave, Stress, Propagation time

## Abstract

The objective of this study is to obtain the propagation velocity of an elastic wave in a loaded isotropic solid and to show the usefulness of the third-order elastic constant in determining properties of practical materials. As is well known, the infinitesimal elastic theory is unable to express the influence of stress on elastic wave propagating in loaded materials. To solve this problem, the authors derive an equation of motion for elastic wave in a finitely deformed state and use the Lagrangian description where the state before deformation is used as a reference, and Murnaghans finite deformation theory for the unidirectional deformed isotropic solid. Ordinary derivatives were used for the mathematical treatment and although the formulas are long the content is simple. The theory is applied to the measurement of the third-order elastic constants of common steels containing carbon of 0.22 and 0.32 wt%. Care is taken in preparing specimens to precise dimensions, in properly adhering of transducer to the surface of the specimen, and in having good temperature control during the measurements to obtain precise data. As a result, the stress at various sites in the structural materials could be estimated by measuring the elastic wave propagation times. The results obtained are graphed for illustration.

## Background

There is little discussion of the practical application of the third-order elastic constants from the viewpoint of engineering. The third-order elastic constants and its mathematical procedure of practical materials were first reported by Hughes and Kelly (1953), however their mathematical treatments were difficult to understand.

In this paper we introduced the formulas to show the relationship between the velocity of the elastic wave propagation and the stresses under the assumption that the elastic waves propagate in the unidirectional loaded isotropic materials. Cast in plain mathematics, we use Murnaghans finite elastic theory (Murnaghan 1951) combined with the Lagrangian description for a simpler description.

Three coordinate systems were used to treat the elastic waves in the finitely deformed solid; the first coordinate system corresponds to the non-deformed state, the second to the statically finitely deformed state, and the third to the state where an infinitesimal dynamical deformation is superposed on the finite deformation of the second state.

The application of our formulas in estimating unknown stresses were tested by measuring the ratio of change in propagation time to stress for the common steels. The points to be elaborated are based on followings;

(A) Use of the Lagrangian description for an unloaded non-deformed isotropic object;
(B) Propagation of an elastic wave in a finitely deformed object loaded and stressed in the uniaxial direction.
(C) Derivation of the propagation velocity of the elastic wave in a loaded object from the viewpoint of Murnaghans’s finite deformation theory;
(D) Application of the third-order elastic constants to the stress measurement.

## Coordinates

1. The coordinates of the unloaded and non-deformed isotropic object are denoted by $a_1, a_2,a_3$, and expressed as $a_{i}$, here $i =1, 2, 3.$
2. The coordinates in the statically and elastically deformed state are denoted by $X_1, X_2, X_3$, then expressed as $X_{i}$, and the displacements are denoted by $U_1$, $U_2$, $U_3$, then expressed similarly by $U_i$, here $U_1=X_1-a_1$, $U_2=X_2-a_2$, $U_3=X_3-a_3$, so $U_i=X_i-a_i$
3. The coordinates of the statically deformed state superposed by the elastic wave are denoted similarly by $x_i$. The infinitesimal displacement of elastic wave are denoted by $u_1, u_2, u_3$, then expressed by $u_i$, here $u_1=x_1-X_1, u_2=x_2-X_2, u_3=x_3-X_3$, so $u_i=x_i-X_i$
4. When the coordinate $a_i$ in the non-deformed state changes to the coordinate $U_i$ of the finitely deformed state by applying load and further applying the infinitesimal displacement of $u_i$, the coordinates $x_i$ are expressed as formula (1) as
  1 $$\begin{aligned} x_i=a_i+U_i+u_i=X_i+u_i, X_i=a_i+U_i \end{aligned}$$

## Strain

Total strain $\bar{\eta }_{ij}$ superposed by the infinitesimal strain of elastic wave $\hat{\eta _{ij}}$ on the static strain $\eta _{ij}$ is expressed as formula (2) as

2 $$\begin{aligned} \bar{\eta }_{ij} = \frac{1}{2}\left( \frac{\partial x_\alpha }{\partial a_i}\cdot \frac{\partial x_\alpha }{\partial a_j} - \delta _{ij} \right) \end{aligned}$$

3 $$\begin{aligned} \bar{\eta }_{ij}=\eta _{ij}+\hat{\eta }_{ij} \end{aligned}$$

where subscripts $i$, $j$, $\alpha$ and $\beta$ take 1, 2, and 3. We assume that Greek lettered subscripts are summed indices, but Roman lettered subscripts are not summed. $\delta _{ij}$: Kronecker’s delta, when $i=j$, $\delta _{ij}=1$, and $\textit{i}\ne \textit{j}$, $\delta _{ij}= 0$

Using formula (1), formula (2) is rewritten as

4 $$\begin{aligned} \bar{\eta }_{ij}&= \frac{1}{2}\left( \frac{\partial x_\alpha }{\partial a_i}\frac{\partial x_\alpha }{\partial a_j} - \delta _{ij}\right) \nonumber \\&= \frac{1}{2}\left( \left( \frac{\partial (a_\alpha +U_\alpha )}{\partial a_i}+\frac{\partial u_\alpha }{\partial a_i}\right) \cdot \left( \frac{\partial (a_\alpha + U_\alpha )}{\partial a_j} + \frac{\partial u_\alpha }{\partial a_j}\right) -\delta _{ij}\right) \nonumber \\&= \frac{1}{2}\left( \frac{\partial (a_\alpha +U_\alpha )}{\partial a_i}\cdot \frac{\partial ( a_\alpha +U_\alpha )}{\partial a_j}-\delta _{ij}\right) \nonumber \\&\quad +\frac{1}{2}\left( \frac{\partial (a_\alpha +U_\alpha )}{\partial a_i}\cdot \frac{\partial u_\alpha }{\partial a_j}+\frac{\partial (a_\alpha + U_\alpha )}{\partial a_j} \cdot \frac{\partial u_\alpha }{\partial a_i} + \left( \frac{\partial u_\alpha }{\partial a_i}\cdot \frac{\partial u_\alpha }{\partial a_j} \right) \right) \end{aligned}$$

The first term in formula (4) corresponds to $\eta _{ij}$ of formula (3) whereas the second term corresponds to $\hat{\eta }_{ij}$. After this, the derivation of the formula stands on the following assumptions,

1. Terms equal or higher than second order in the infinitesimal displacement gradient ${\partial u_i}/{\partial X_j}$ can be neglected.
2. Terms equal or higher than third-order products of ${\partial u_i}/{\partial X_j}$ and the finite displacement gradient ${\partial U_i}/{\partial a_j}$ can be neglected.

Next, $\hat{\eta }_{ij}$ in formula (4) is written as

5 $$\begin{aligned} \hat{\eta }_{ij}&= \frac{1}{2}\biggl [\left( \delta _{\alpha i}+\frac{\partial U_\alpha }{\partial a_i}\right) \cdot \frac{\partial u_\alpha }{\partial X_\beta }\cdot \frac{\partial X_\beta }{\partial a_j} \nonumber \\&\quad +\left( \delta _{\alpha j}+\frac{\partial U_\alpha }{\partial a_j}\right) \cdot \frac{\partial u_\alpha }{\partial X_\beta }\cdot \frac{\partial X_\beta }{\partial a_i}+ \frac{\partial u_\alpha }{\partial X_\beta } \cdot \frac{\partial X_\beta }{\partial a_i}\cdot \frac{\partial u_\alpha }{\partial X_\gamma }\cdot \frac{\partial X_\gamma }{\partial a_j}\biggr ] \nonumber \\&\approx \frac{1}{2}\biggl [\left( \delta _{\alpha i}+\frac{\partial U_\alpha }{\partial a_i} \right) \left( \delta _{\beta j} + \frac{\partial U_\beta }{\partial a_j}\right) + \left( \delta _{\alpha j} + \frac{\partial U_\alpha }{\partial a_j}\right) \left( \delta _{\beta i} + \frac{\partial U_\beta }{\partial a_i}\right) \biggr ]\frac{\partial u_\alpha }{\partial X_\beta } \end{aligned}$$

In the above formula (5), the two terms in the first factor are symmetric with regard to interchanging *i* and *j*; then interchanging $$\alpha$$$$\rightarrow$$$$\beta$$, and $$\beta$$$$\rightarrow$$$$\alpha$$ in the second factor, and similarly changing the subscript of the Greek alphabet in the $${\partial u_\alpha }/{\partial X_\beta }$$ yields

6 $$\begin{aligned} \hat{\eta }_{ij}= \frac{1}{2}\left( \left( \delta _{\alpha i} + \frac{\partial U_\alpha }{\partial a_i}\right) \left( \delta _{\beta j} + \frac{\partial U_\beta }{\partial a_j}\right) \right) \left( \frac{\partial u_\alpha }{\partial X_\beta }+\frac{\partial u_\beta }{\partial X_\alpha }\right) \end{aligned}$$

Accordingly $\hat{\eta }_{ij}= \hat{\eta }_{ji}$, and the infinitesimal strain is symmetric.

See Appendix A about the calculations of $\hat{\eta }_{11}$, $\hat{\eta }_{22}$, and $\hat{\eta }_{33}$.

## Stresses

### Static stress $T_{ij}$

Following Murnaghan’s theory, static stress $T_{ij}$ is written as

7 $$\begin{aligned} T_{ij}=J_{i\alpha }\frac{\partial (\rho _0 \phi )}{\partial \eta _{\alpha j}} \end{aligned}$$

where $J_{i\alpha }$ is the Jacobian matrix, $\rho _0\phi$ the free energy per unit volume of deformed isotropic solid, and $\rho _0$ the density of isotropic solid in non-deformed state. The free energy per unit volume can be written in terms of the strain invariants $I_1, I_2, I_3$

8 $$\begin{aligned} \rho _0\phi =A_0I_1+\frac{(\lambda +2\mu )}{2}{I_1}^2 - 2\mu I_2+\frac{(\ell +m)}{3}{I_1}^3-2mI_1I_2+nI_3 \end{aligned}$$

and hence its derivatives with respect to the static strain coefficients are

9 $$\begin{aligned} \frac{\partial (\rho _0 \phi )}{\partial \eta _{ij}}&= \left( (\lambda +2\mu ) I_1+(\ell +2m){I_1}^2-2mI_2\right) \frac{\partial I_1}{\partial \eta _{ij}} \nonumber \\&\quad -(\mu +mI_1)\frac{\partial I_2}{\partial \eta _{ij}}+n\frac{\partial I_3}{\eta _{ij}} \end{aligned}$$

where $$A_0 =0,$$$$\lambda$$ and $$\mu$$ are the Lamé constants, $$l, m, n$$ are Murnaghan’s third-order elastic constants, and the strain invariants are defined by

$$\begin{aligned} I_1=\eta _{11}+\eta _{22}+\eta _{33} = \eta _{\beta \alpha }\delta _{\beta \alpha } \end{aligned}$$

10 $$\begin{aligned} I_2&= \left| \begin{array}{rr} \eta _{22} &{} \eta _{23} \\ \eta _{32} &{} \eta _{33} \end{array} \right| + \left| \begin{array}{rr} \eta _{33} &{} \eta _{31} \\ \eta _{13} &{} \eta _{11} \end{array} \right| + \left| \begin{array}{rr} \eta _{11} &{} \eta _{12} \\ \eta _{21} &{} \eta _{22} \end{array} \right| \nonumber \\&=\frac{1}{2}(\eta _{\alpha \alpha }\eta _{\beta \beta }-\eta _{\alpha \beta }\eta _{\beta \alpha }) \end{aligned}$$

11 $$\begin{aligned} I_3= \left| \begin{array}{rrr} \eta _{11} &{} \eta _{12} &{} \eta _{13} \\ \eta _{21} &{} \eta _{22} &{} \eta _{23} \\ \eta _{31} &{} \eta _{32} &{} \eta _{33} \end{array} \right| =\frac{1}{2}\eta _{\alpha \alpha }(\eta _{\gamma \gamma }\eta _{\beta \beta } -\eta _{\alpha \beta }\eta _{\beta \alpha }), \quad (\alpha \ne \beta \ne \gamma ) \end{aligned}$$

The derivatives of the above invariants of $I_1, I_2$ and $I_3$ are given as,

12 $$\begin{aligned} \frac{\partial I_1}{\partial \eta _{11}}&=1, \frac{\partial I_1}{\partial \eta _{23}}=0, \frac{\partial I_2}{\partial \eta _{11}}=\eta _{22} + \eta _{33}=I_1-\eta _{11}, \nonumber \\ \frac{\partial I_2}{\partial \eta _{23}} &= -\eta _{32}, \frac{\partial I_3}{\partial \eta _{11}}=\eta _{22}\eta _{33}-\eta _{23}\eta _{32}, \end{aligned}$$

13 $$\begin{aligned} \frac{\partial I_3}{\partial \eta _{23}}=\eta _{12}\eta _{31}-\eta _{11}\eta _{32}=- \left| \begin{array}{rr} \eta _{11} &{} \eta _{12} \\ \eta _{31} &{} \eta _{32} \end{array} \right| \end{aligned}$$

Thus the derivative of the free energy $\rho _0\phi$ with respect to $\eta _{11}$, for example, becomes

14 $$\begin{aligned} \frac{\partial (\rho _0\phi )}{\partial \eta _{11}}&=(\lambda +2\mu )I_1+(\ell +2m)I_1^2-2mI_2 \nonumber \\&\quad -2(\mu +mI_1)(I_1-\eta _{11})+n(\eta _{22}\eta _{33}-\eta _{23}\eta _{32}) \end{aligned}$$

See Appendix B on the derivative with respect to $\eta _{22}$, $\eta _{33}$, $\eta _{23}$, $\eta _{31}$, $\eta _{12}$.

### Infinitesimal stress of elastic wave ${\hat{T}_{ij}}$

The total stress ${\bar{T}}_{ij}$ is defined as the sum of the infinitesimal stress ${\hat{T}}_{ij}$ and the static stress $T_{ij}$, that is,

15 $$\begin{aligned} {\bar{T}}_{ij}=(T_{ij} + {\hat{T}}_{ij}) \end{aligned}$$

Using formula (7) and replacing $\rho$ for $\rho _0 \phi$, formula (15) is rewritten as

16 $$\begin{aligned} \bar{T}_{ij}&=\bar{J}_{i \alpha }\Bigl (\frac{ \partial \bar{\phi }}{\partial \eta _{\alpha j}} \Bigr ) \nonumber \\&=(J_{i \alpha } + \hat{J}_{i \alpha })\Bigl (\frac{\partial \phi }{\partial \eta _ {\alpha j}} + \frac{\partial \hat{\phi }}{\partial \eta _{\alpha j}}\Bigr ) \nonumber \\&= J_{i \alpha }\frac{\partial \phi }{\partial \eta _{\alpha j}} + \hat{J}_{i\alpha }\frac{\partial \phi }{\partial \eta _{\alpha j}} + J_{i \alpha }\frac{\partial \hat{\phi }}{\partial \eta _{\alpha j}} \nonumber \\&\quad +\hat{J}_{i \alpha }\frac{\partial \hat{\phi }}{\partial \eta _{\alpha j}} \end{aligned}$$

17 $$\begin{aligned} \hat{T} _{ij}=\hat{J}_{i \alpha }\frac{\partial \phi }{\partial \eta _{\alpha j}} + ( J_{i \alpha } + \hat{J}_{i \alpha })\frac{\partial \hat{\phi }}{\partial \eta _{\alpha j}} \end{aligned}$$

Here the elements of the Jacobian matrix are expressed as

18 $$\begin{aligned} \bar{J}_{i \alpha }&=\frac{\partial x_i}{\partial {a_i}}, \nonumber \\ J_{i \alpha } &=\frac{\partial X_i}{\partial a_\alpha } =\frac{\partial (a_i + U_i)}{\partial a_\alpha } \nonumber \\ &=\delta _{i \alpha }+\frac{\partial U_i}{\partial a_\alpha }, \nonumber \\ \hat{J}_{i \alpha } &=\bar{J}_{i \alpha } - J_{i \alpha } \nonumber \\ &=\frac{\partial x_i}{\partial a_\alpha }-\frac{\partial X_i}{\partial a_\alpha } \nonumber \\ &=\frac{\partial u_i}{\partial a_\alpha } \end{aligned}$$

Using the above formula (18),

19 $$\begin{aligned} &J_{11}=\frac{\partial X_1}{\partial a_1} = \left( 1 + \frac{\partial U_1}{\partial a_1}\right),\quad J_{23} = \frac{\partial X_2}{\partial a_3} = \frac{\partial U_2}{\partial a_3}, \nonumber \\ &\hat{J}_{11} = \frac{\partial x_1}{\partial a_1} - \frac{\partial X_1}{\partial a_1} = \frac{\partial u_1}{\partial a_1}, \quad \hat{J}_{22}=\frac{\partial u_2}{\partial a_2},\quad \hat{J}_{33} =\frac{\partial u_3}{\partial a_3} \end{aligned}$$

20 $$\begin{aligned} \hat{J}_{23} = \frac{\partial x_2}{\partial a_3} - \frac{\partial X_2}{\partial a_3} = \frac{\partial u_2}{\partial a_3},\quad \hat{J}_{31}=\frac{\partial u_3}{\partial a_1},\quad \hat{J}_{12} =\frac{\partial u_1}{\partial a_2} \end{aligned}$$

See Appendix C about expressions for the derivatives of ${\partial u_1}/{\partial a_1}$, ${\partial u_1}/{\partial a_2}$, and ${\partial u_1}/{\partial a_3}$.

21 $$\begin{aligned} \hat{T}_{ij} \approx \hat{J}_{i \alpha } \frac{\partial \phi }{\partial \eta _{\alpha j}} + J_{i \alpha }\frac{\partial \hat{\phi }}{\partial \eta _{\alpha j}} \end{aligned}$$

Setting $\alpha =1$, and $j=1$ in the ${\partial \hat{\phi }}/{\partial \eta _{\alpha j}}$, yield

22 $$\begin{aligned} \frac{\partial \hat{\phi }}{\partial \eta _{11}} = \left( \frac{\partial \bar{\phi }}{\partial \eta _{11}}\right) -\frac{\partial \phi }{\partial \eta _{11}} \end{aligned}$$

Expanding formula (22) using formula (14) then gives

23 $$\begin{aligned} \frac{\partial \hat{\phi }}{\partial \eta _{11}} &=\lambda \bar{I}_1 + \ell {\bar{I}_1}^2 -2m \bar{I}_2 +2 \bar{\eta }_{11} (\mu + m \bar{I}_1) +n( \bar{\eta }_{22}\bar{\eta }_{33}-\bar{\eta }_{23} \bar{\eta }_{32}) \nonumber \\&\quad -\lambda I_1 -\ell {I_1}^2 +2m I_2-2\eta _{11} (\mu +m I_1) -n(\eta _{22}\eta _{33}-\eta _{23}\eta _{32}) \nonumber \\ &\approx ( \lambda +2\mu +2\ell I_1+4m \eta _{11} )\hat{\eta }_{11}+(\lambda +2\ell I_1-(2m-n)\eta _{33})\hat{\eta }_{22} \nonumber \\&\quad +(\lambda +2\ell I_1-(2m-n)\eta _{22})\hat{\eta }_{33}+(2m-n)(\eta _{23}\hat{\eta }_{32}+\eta _{32}\hat{\eta }_{23}) \nonumber \\&\quad +2m(\eta _{13}\hat{\eta }_{31}+\eta _{31}\hat{\eta }_{13}+\eta _{12}\hat{\eta }_{21} +\eta _{21}\hat{\eta }_{12}) \end{aligned}$$

See Appendix D for a detailed derivation. Other derivatives are similarly obtained

An expression for $\partial {\hat{\phi }}$ / $\partial {\eta _{22}}$ can be obtained through changing subscripts 1$\rightarrow$2, 2$\rightarrow$3, 3$\rightarrow$1 in the formula 23).

The formula of $\partial {\hat{\phi }} /\partial {\eta _{33}}$ can be obtained in a similar manner. Also

24 $$\begin{aligned} \frac{\partial \hat{\phi }}{\partial \eta _{12}} &\approx(2m-n)\eta _{21}\hat{\eta }_{33}+2m\eta _{21}(\hat{\eta }_{11} + \hat{\eta }_{22}) \nonumber \\&\quad + (2\mu +2m I_1 - n\eta _{33}) \hat{\eta }_{21} +n(\eta _{31}\hat{\eta }_{23} + \eta _{23}\hat{\eta }_{31}) \end{aligned}$$

and hence by $1\leftrightarrow 2$

25 $$\begin{aligned} \frac{\partial \hat{\phi }}{\partial \eta _{21}} &\approx(2m-n)\eta _{12}\hat{\eta }_{33}+2m\eta _{12}(\hat{\eta }_{22}+ \hat{\eta }_{11}) \nonumber \\&\quad + (2\mu +2m I_1 - n\eta _{33}) \hat{\eta }_{12} +n(\eta _{32}\hat{\eta }_{13} + \eta _{13}\hat{\eta }_{32}) \end{aligned}$$

Using the above formulas (21) to (25), formula (17) for the infinitesimal stress is rewritten as follows.

26 $$\begin{aligned} \hat{T}_{11} &=\hat{J}_{1 \alpha } \frac{\partial \phi }{\partial \eta _{\alpha 1}}+ J_{1 \alpha } \frac{\partial \hat{\phi }}{\partial \eta _{\alpha 1}} \nonumber \\ &\approx \left( \lambda +2\mu +(\lambda +2\ell )I_1+(3\lambda +8\mu +4m)\eta _1 \right) \ \dot{\eta _1} \nonumber \\&\quad + \left( \lambda +(\lambda +2\ell )I_1+\lambda \eta _2-(\lambda +2m - n) \eta _3\right) \dot{\eta _2} \nonumber \\&\quad +\left( \lambda +(\lambda +2\ell )I_1+\lambda \eta _3-(\lambda +2m-n)\eta _2 \right) \dot{\eta _3} \nonumber \\&\quad +\frac{1}{2}(\lambda +2m-n)\eta _4 \dot{\eta _4} +\frac{1}{2}(2\lambda +3\mu +2m)(\eta _5 \dot{\eta _5} + \eta _6 \dot{\eta _6}) \nonumber \\&\quad + 2\mu \left( \eta _{12}\frac{\partial u_1}{\partial X_2} + \eta _{13}\frac{\partial u_1}{\partial X_3}\right) \end{aligned}$$

See Appendix E for details of the derivation for $\hat{T}_{11}$. Also,

27 $$\begin{aligned} \hat{T}_{22} &\approx \left( (\lambda +2\mu +(\lambda +2\ell )I_1+(3\lambda +8\mu +4m)\eta _2\right) \ \dot{\eta _2} \nonumber \\&\quad + \left( \lambda +(\lambda +2\ell )I_1+\lambda \eta _3-(\lambda +2m-n)\eta _1\right) \dot{\eta _3} \nonumber \\&\quad +\left( \lambda +(\lambda +2\ell )I_1+\lambda \eta _1-(\lambda +2m-n)\eta _3\right) \dot{\eta _1} \nonumber \\&\quad + \frac{1}{2}(\lambda +2m-n)\eta _5 \dot{\eta _5} +\frac{1}{2}(2\lambda +2m+3\mu )(\eta _4 \dot{\eta _4}+\eta _6\dot{\eta _6}) \nonumber \\&\quad + 2\mu \left( \eta _{23}\frac{\partial u_2}{\partial X3} + \eta _{21}\frac{\partial u_2}{\partial X_1}\right) \end{aligned}$$

28 $$\begin{aligned} \hat{T}_{33} & \approx \left( \lambda +2\mu +(\lambda +2\ell )I_1+(3\lambda +8\mu +4m)\eta _3\right) \ \dot{\eta _3} \nonumber \\&\quad + \left( \lambda +(\lambda +2\ell )I_1+\lambda \eta _1 -(\lambda +2m-n)\eta _2\right) \dot{\eta _1} \nonumber \\&\quad +\left( \lambda +(\lambda +2\ell )I_1-(\lambda +2m-n)\eta _1+\lambda \eta _2\right) \dot{\eta _2} \nonumber \\&\quad + \frac{1}{2}(\lambda +2m-n)\eta _6 \dot{\eta _6} +\frac{1}{2}(2\lambda +2m+3\mu )(\eta _4 \dot{\eta _4}+\eta _5\dot{\eta _5}) \nonumber \\&\quad + 2\mu \left( \eta _{31}\frac{\partial u_3}{\partial X_1} + \eta _{32}\frac{\partial u_3}{\partial X_2}\right) \end{aligned}$$

In the expression for $\hat{T}_{ij}$, the subscripts of 1, 2, and 3 for $\eta _\alpha$ and $\acute{\eta _\alpha }$ change cyclically with 1 $\rightarrow$ 2, 2 $\rightarrow$ 3, 3 $\rightarrow$ 1. Similarly, indices 4, 5, 6 change cyclically like 4 $\rightarrow$ 5, 5 $\rightarrow$ 6, 6 $\rightarrow$ 4.

For $\eta _{\alpha \beta }$ and $\partial {u_\alpha } /\partial {X_\beta }$, the subscripts change cyclically as well with 1 $\rightarrow$ 2, 2 $\rightarrow$ 3, 3 $\rightarrow$ 1. Hence from

29 $$\begin{aligned} \hat{T}_{23} &=\hat{J}_{2 \alpha }\frac{\partial \phi }{\partial \eta _{\alpha 3}}+ J_{2 \alpha } \frac{\partial \hat{\phi }}{\partial \eta _{\alpha 3}} \nonumber \\ &=2\mu \eta _{31}\frac{\partial u_2}{\partial X_1} +( \lambda I_1+2\mu \eta _{33}) \frac{\partial u_2}{\partial X_3} \nonumber \\&\quad + \frac{1}{2}\Bigl ((\lambda +2m-n)\dot{\eta _1} +\frac{1}{2}(\lambda +4\mu +2m)\dot{\eta _2} +\frac{1}{2}(\lambda +4\mu +2m)\dot{\eta _3}\Bigr )\eta _4 \nonumber \\&\quad +\frac{1}{2}\left (2\mu +2(\mu +m)I_1-(2\mu +n)\eta _1+2\mu \eta _2\right )\dot{\eta _4} \nonumber \\&\quad +\frac{1}{4}(4\mu +n)\eta _6\dot{\eta _5}+\frac{1}{4}(2\mu +n)\eta _5\dot{\eta _6} \end{aligned}$$

we obtain

30 $$\begin{aligned} \hat{T}_{32}&=(\lambda I_1+2\mu \eta _{22})\frac{\partial u_3}{\partial X_2}+2\mu \eta _{21}\frac{\partial u_3}{\partial X_1} \nonumber \\&\quad +\frac{1}{2}\Bigl ((\lambda +2m-n)\dot{\eta _1} +\frac{1}{2}(\lambda +2m+4\mu )\dot{\eta _2}+\frac{1}{2}(\lambda +4\mu +2m)\dot{\eta _3}\Bigl )\eta _4 \nonumber \\&\quad + \frac{1}{2}\Bigl (2\mu +2(\mu +m)I_1-(2\mu +n)\eta _1+2\mu \eta _3\Bigl )\dot{\eta _4} \nonumber \\&\quad +\frac{1}{4}(4\mu +n)\eta _5\dot{\eta _6} + \frac{1}{4}(2\mu +n)\eta _6\dot{\eta _5} \end{aligned}$$

See 13 about induction process of $\hat{T}_{11}$.

31 $$\begin{aligned} \hat{T}_{31} &=(\lambda I_1+2\mu \eta _{11})\frac{\partial u_3}{\partial X_1} +2\mu \eta _{12}\frac{\partial u_3}{\partial X_2} \nonumber \\&\quad +\frac{1}{2}(\lambda +2\mu +2m-n)\dot{\eta _2} + \frac{1}{2}(\lambda +4\mu +2m)\dot{\eta _3} \nonumber \\&\quad +\frac{1}{2}(\lambda +4\mu +2m-n)\dot{\eta _1} \nonumber \\&\quad +\frac{1}{2}\Bigl (2\mu +2(\mu +m)I_1-(2\mu +n)\eta _2+2\mu \eta _3\Bigl )\dot{\eta _5} \nonumber \\&\quad +\frac{1}{4}(4\mu +n)\eta _4\dot{\eta _6}+ \frac{1}{4}(2\mu +n)\eta _6\dot{\eta _4} \end{aligned}$$

$\hat{T}_{13}$ can be obtained from formula (31) for $\hat{T}_{31}$ by substituting elements as follows:

$${\partial u_3}/{\partial X_1}\rightarrow {\partial u_1}/{\partial X_3},\qquad {\partial u_3}/{\partial X_2} \rightarrow {\partial u_1}/{\partial X_2},\qquad\eta _4\acute{\eta _6}\rightarrow \eta _6\acute{\eta _4},\qquad \eta _6\acute{\eta _4}\rightarrow \eta _4\acute{\eta _6}$$

and retaining the elements within the parentheses as these are unaffected by the interchange in the formula of $\hat{T}_{13}$.

$\hat{T}_{12}$ is expressible as

32 $$\begin{aligned} \hat{T}_{12}&=(\lambda I_1+2\mu \eta _{22}) \frac{\partial u_1}{\partial X_2} + 2\mu \eta _{23}\frac{\partial u_1}{\partial X_3} \nonumber \\&\quad + \frac{1}{2}(\lambda +2\mu +2m-n)\dot{\eta _3}+\frac{1}{2}(\lambda +4\mu +2m)\dot{\eta _1} \nonumber \\&\quad +\frac{1}{2}(\lambda +4\mu +2m-n)\dot{\eta _2} \nonumber \\&\quad +\frac{1}{2}\Bigl (2\mu +2(\mu +m)I_1-(2\mu +n)\eta _3+2\mu \eta _1\Bigl )\dot{\eta _6} \nonumber \\&\quad +\frac{1}{4}(4\mu +n)\eta _5\dot{\eta _4} +\frac{1}{4}(2\mu +n)\eta _4\dot{\eta _5} \end{aligned}$$

from which $\hat{T_{21}}$ can be obtained by changing elements ${\partial u_1}/{\partial X_2}\rightarrow {\partial u_2}/{\partial X_1}$, ${\partial u_1}/{\partial X_3} \rightarrow {\partial u_2}/{\partial X_3}$, $\eta _5\acute{\eta _4}\rightarrow \eta _4\acute{\eta _5}$, $\eta _4\acute{\eta _5}\rightarrow \eta _5\acute{\eta _4}$, while retaining the elements in the parentheses as these are unaffected by the index interchanges.

### Propagation velocity of elastic wave to the direction of static uniaxial stress

The infinitesimal displacement of an elastic wave $u_i$ is expressed as

33 $$\begin{aligned} u_i=A\;exp\;\mathbf {i}\;(\omega t-\kappa X_i) \end{aligned}$$

where A is the amplitude, $\omega$ the angular frequency, $\kappa$ the wave number, and $\mathbf {i}$ the imaginary unit.

The equation of motion for an elastic wave is written as

34 $$\begin{aligned} \rho _0 \frac{\partial ^2 u_i}{\partial t^2}= \frac{\partial \hat{T}_{i \alpha }}{\partial a_\alpha } = \frac{\partial \hat{T}_{i \alpha }}{\partial X_\beta }\frac{\partial X_\beta }{\partial a_\alpha } \end{aligned}$$

The various expansions of formula (34) are given as (A) to (E) as bellow:

(A) For longitudinal wave
  35 $$\begin{aligned} \rho _0 \frac{\partial ^2 u_1}{\partial t^2}= \frac{\partial \hat{T}_{1 \alpha }}{\partial X_\beta }\frac{\partial X_\beta }{\partial a_\alpha } =\frac{\hat{T}_{1 1}}{\partial X_\beta }\frac{\partial X_\beta }{\partial a_1}+\frac{\hat{T}_{12}}{\partial X_\beta }\frac{\partial X_\beta }{\partial a_2} +\frac{\hat{T}_{13}}{\partial X_\beta }\frac{\partial X_\beta }{\partial a_3} \end{aligned}$$
  ${\partial U_i}/{\partial a_j}=0$, $(i\ne j)$ The expansion of the above formula (35) is
  36 $$\begin{aligned} \rho _0 \frac{\partial ^2 u_1}{\partial t^2} = \frac{\partial \hat{T}_{11}}{\partial X_1}\left( 1+\frac{\partial U_1}{\partial a_1}\right) + \frac{\partial \hat{T}_{12}}{\partial X_2}\left( 1+\frac{\partial U_2}{\partial a_2}\right) + \frac{\partial \hat{T}_{13}}{\partial X_3}\left( 1+\frac{\partial U_3}{\partial a_3}\right) \end{aligned}$$
  $$\begin{aligned} u_1 = A \;\ exp\; \mathbf {i}\; (\omega t -\kappa X_1),\quad u_2= u_3 = 0 \quad \dot{\eta _2} = \dot{\eta _3} = \dot{\eta _4} = \dot{\eta _5} = \dot{\eta _6} = 0 \end{aligned}$$
  Accordingly,
  37 $$\begin{aligned} \rho _0\frac{\partial ^2 u_1}{\partial t^2}=(1+\eta _1) \frac{\partial }{\partial X_1}\Bigl ( \lambda + 2\mu + (\lambda +2\ell )I_1+(3\lambda +8\mu +4m)\eta _1\Bigl )\dot{\eta _1} \end{aligned}$$
  From the expressions for ${\partial ^2 u_1}/{\partial t^2}$, ${\partial }/{\partial X_1}$, and $\dot{\eta _1}$ of the above formula (37),
  38 $$\begin{aligned} \frac{\partial ^2 u_1}{\partial t^2} / \frac{\partial \dot{\eta _1}}{\partial X_1} = (\omega /k)^2 = V^2_{11} \end{aligned}$$
  and hence
  39 $$\begin{aligned} \rho _0 V^2_{11}&=(1+\eta _1)\Bigl (\lambda +2\mu +(\lambda +2\ell )I_1+(3\lambda +8\mu +4m)\eta _1\Bigr ) \nonumber \\ & \approx \lambda +2\mu +\frac{T_{11}}{E}\Bigl (\lambda +2\mu +\lambda +2\ell +3\lambda +8\mu +4m-2\nu (\lambda +2\ell )\Bigr ) \nonumber \\ &= \lambda +2\mu +\frac{T_{11}}{E}\Bigl (5\lambda +10\mu +2\ell +4m-2\nu (\lambda +2\ell )\Bigr ) \end{aligned}$$
  where the term quadratic in strain, $\eta ^2_1$ is neglected, and we have used
  40 $$\begin{aligned} \eta _1=\frac{\partial U_1}{\partial a_1} =\frac{T_{11}}{E},\quad \frac{\eta _2}{\eta _1}= \frac{\eta _3}{\eta _1}=-\nu \end{aligned}$$
  where $\nu$ is Poisson’s ratio, and E is Young’s modulus
(B) For transverse wave
  41 $$\begin{aligned} \rho _0\frac{\partial ^2 u_2}{\partial t^2}=\left( 1+\frac{\partial U_1}{\partial a_1}\right) \frac{\partial \hat{T}_{21}}{\partial X_1}+ \left( 1+\frac{\partial U_2}{\partial a_2}\right) \frac{\partial \hat{T}_{22}}{\partial X_2}+ \left( 1+\frac{\partial U_3}{\partial a_3}\right) \frac{\partial \hat{T}_{23}}{\partial X_3} \end{aligned}$$
  $$\begin{aligned} u_2= A\; exp\; \mathbf { i }(\omega t - k X_1), \quad u_1=u_3 = 0, \quad \dot{\eta _6} \ne 0\end{aligned}$$
  the others are 0,
  $$\begin{aligned} \frac{\partial U_i}{\partial a_j} = 0, i\ne j \end{aligned}$$
  then
  42 $$\begin{aligned} \rho _0 \frac{\partial ^2 u_2}{\partial t^2} &=(1+\eta _1) ((\lambda I_1+2\mu \eta _{11}) \nonumber \\&\quad +\frac{1}{2} (2\mu +2(\mu +m)I_1 -(2\mu +n)\eta _3+2\mu \eta _2 ))\frac{\partial ^2 u_2}{\partial X^2_1} \end{aligned}$$
  43 $$\begin{aligned} \frac{\partial ^2 u_2}{\partial t^2} / \frac{\partial ^ 2u_2}{\partial X^2_1} = (\omega /k)^2 = V^2_{12} \end{aligned}$$
  44 $$\begin{aligned} \rho _0 V^2_{12} &=(1+\eta _1)\Bigl (\mu +(\lambda +\mu +m)I_1+2\mu \eta _{11}+\mu \eta _2-\frac{1}{2}(2\mu +n)\eta _3 \Bigr ) \nonumber \\ &=\mu +\frac{T_{11}}{E}\Bigl (\mu +\lambda +\mu +m+2\mu -\nu \left( 2\lambda +2\mu +2m+\mu -\mu -\frac{n}{2}\right) \Bigr ) \nonumber \\ &=\mu +\frac{T_{11}}{E}\Bigl (\lambda +4\mu +m-\nu \left( 2\lambda +2\mu +2m-\frac{n}{2}\right) \Bigr ) \end{aligned}$$
  where the term of $\eta ^2_1$ is also neglected in a similar way to formula (39).
(C) For transverse wave
  45 $$\begin{aligned} \rho _0 \frac{\partial ^2 u_1}{\partial t^2} &=\left ( 1+\frac{\partial U_1}{\partial a_1}\right )\frac{\partial \hat{T}_{11}}{\partial X_1} + \left ( 1+\frac{\partial U_2}{\partial a_2}\right )\frac{\partial \hat{T}_{12}}{\partial X_2} + \left ( 1+\frac{\partial U_3}{\partial a_3}\right )\frac{\partial \hat{T}_{13}}{\partial X_3} \nonumber \\ u_1 &= A\; exp\; \mathbf { i }( \omega t- k X_2), \quad u_2 =u_3 = 0 \end{aligned}$$
  46 $$\begin{aligned} \rho _0 \frac{\partial ^2 u_1}{\partial t^2} = ( 1+\eta _2)\Bigl (\lambda I_1 +2\mu \eta _2+ \mu + (\mu +m) I_1-\left( \mu + \frac{n}{2}\right) \eta _3 +\mu \eta _1\Bigr )\frac{\partial u_1}{\partial X_3} \end{aligned}$$
  47 $$\begin{aligned} \rho _0 V^2_{21} &= ( 1 + \eta _2)\left (\mu + ( \lambda +\mu +m) I_1 +\mu \eta _1 +2\mu \eta _2 -\left( \mu +\frac{n}{2}\right) \eta _3 \right ) \nonumber \\ &=\mu + \frac{T_{11}}{E} \left (\lambda +\mu +m-\nu \left( 2\lambda +4\mu +2m-\frac{n}{2}\right) \right ) \end{aligned}$$
(D) For longitudinal wave
  48 $$\begin{aligned} &\rho _0 \frac{\partial ^2 u_2}{\partial t^2} =\Bigl ( 1+\frac{\partial U_1}{\partial a_1}\Bigr )\frac{\partial \hat{T}_{21}}{\partial X_1} + \Bigl ( 1+\frac{\partial U_2}{\partial a_2}\Bigr )\frac{\partial \hat{T}_{22}}{\partial X_2} + \Bigl ( 1+\frac{\partial U_3}{\partial a_3}\Bigr )\frac{\partial \hat{T}_{33}}{\partial X_3} \nonumber \\& \qquad u_2 = A\; exp\; \mathbf { i }( \omega t- k X_2), \quad u_1 =u_3 = 0 \end{aligned}$$
  49 $$\begin{aligned} \rho _0 \frac{\partial ^2 u_2}{\partial t^2} = ( 1+\eta _2)\Bigl (\lambda +2\mu + (\lambda +2\ell ) I_1+(3\lambda +8\mu +4m)\eta _2 \Bigr )\frac{\partial ^2 u_2}{\partial X^2_2} \end{aligned}$$
  50 $$\begin{aligned} \rho _0 V^2_{22} &=\lambda + 2\mu + \frac{T_{11}}{E}\left (\lambda + 2\ell -\nu (\lambda +2\mu +2\lambda +4\ell +3\lambda +8\mu +4m) \right ) \nonumber \\ &=\lambda + 2\mu + \frac{T_{11}}{E} \left (\lambda +2\ell -\nu (6\lambda +10\mu +4\ell +4m)\right ) \end{aligned}$$
(E) For transverse wave
  51 $$\begin{aligned}& \rho _0 \frac{\partial ^2 u_3}{\partial t^2} =\left ( 1+\frac{\partial U_1}{\partial a_1}\right )\frac{\partial \hat{T}_{31}}{\partial X_1} + \left ( 1+\frac{\partial U_2}{\partial a_2}\right )\frac{\partial \hat{T}_{32}}{\partial X_2} + \left ( 1+\frac{\partial U_3}{\partial a_3}\right )\frac{\partial \hat{T}_{33}}{\partial X_3} \nonumber \\ &\qquad u_3 = A \;exp\; \mathbf { i }( \omega t- k X_2), \quad u_1 =u_2 = 0 \end{aligned}$$
  52 $$\begin{aligned} \rho _0 \frac{\partial ^2 u_3}{\partial t^2} = ( 1+\eta _2)\Bigl (\lambda I_1 +2\mu \eta _2+ \mu + (\mu +m) I_1-\left( \mu + \frac{n}{2}\right) \eta _1 +\mu \eta _3\Bigr )\frac{\partial ^2 u_3}{\partial X^2_2} \end{aligned}$$
  53 $$\begin{aligned} \rho _0 V^2_{23} &=( 1 + \eta _2)\left (\mu + ( \lambda +\mu +m) I_1 -\left( \mu +\frac{n}{2}\right) \eta _1+2\mu \eta _2 +\mu \eta _3 \right ) \nonumber \\ &=\mu +\frac{T_{11}}{E}\left (\lambda +\mu +m-\mu -\frac{n}{2}-\nu (\mu +2\lambda +2\mu +2m+2\mu +\mu )\right ) \nonumber \\ &=\mu + \frac{T_{11}}{E} \left (\lambda +m-\frac{n}{2}-\nu (2\lambda +6\mu +2m) \right ) \end{aligned}$$

## Measurement of the propagation velocity of elastic waves and the third-order elastic constants

**Figure 1:**
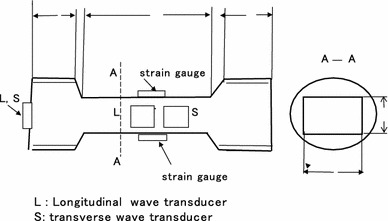
Specimen.

Figure 1 gives a diagram of the axial cross-section through a specimen for tensile testing. In its fabrication, we had to take special care in mixing the raw materials, melting, casting, annealing, and precision working to fix the final form. As stress in the gripping regions is complicated, and should be eliminated, two specimens with identical grip sizes but different gauge lengths were prepared (Takahashi and Motegi 1987).

A transducer was attached at the face of the long axis of the specimen fixed with a chuck with the lead wire. The transducer, a 2–5 MHz PZT, was used for both longitudinal and transverse waves in our experiment. The adhesion conditions between transducer and specimen produced an influence on the accuracy of measurements. The room temperature was kept constant during the measurement. In the above formulas (A)–(E) in the Sect. (4.3), the propagation velocity of elastic waves depends on factor ${T_{11}}/{E}$ and the second or the third-order elastic modulus.

54 $$\begin{aligned} V_{ij} = V_0\left( 1+\alpha _{ij}\frac{T_{11}}{E}\right) \end{aligned}$$

where $\alpha _{ij}$ is the strain dependency coefficient. Thus, for example

55 $$\begin{aligned} V^2_{11} = V^2_0 \left( 1+ \alpha _{11} \frac{T_{11}}{E}\right) ^2\approx V^2_0 \left( 1+2\alpha _{11} \frac{T_{11}}{E}\right) \end{aligned}$$

Without load, $V_0^2 =(\lambda +2\mu )/ {\rho _0}$. If $\alpha _{11}$ is written as in formula (56), then using $V_{11}$ from formula (39),

56 $$\begin{aligned} \alpha _{11} = \frac{1}{2(\lambda +2\mu )}(5\lambda +10\mu +2\ell +4m-2\nu (\lambda +2\ell )) \end{aligned}$$

Similarly $\alpha _{22}$ is written as in formula (57). Then letting $i=j=2$ and using $V_{22}$ as in formula (50), we have

57 $$\begin{aligned} \alpha _{22} = \frac{1}{2(\lambda +2\mu )}(\lambda +2\ell -\nu (6\lambda +10\mu +4\ell +4m) ) \end{aligned}$$

The values of $\alpha _{11}$ and $\alpha _{22}$ can be obtained from measuring $V_{ij}$, $V_0$ and strain in the formula (54). The value of $\alpha _{ij}$ is necessary to obtain the third-order elastic constants. From the obtained $\alpha _{11}$, $\alpha _{22}$, $\alpha _{23}$, and Lame’s constants $\lambda$, $\mu$, and Poisson’s ratio $\nu$, Murnaghan’s third order elastic constants are given as:

58 $$\begin{aligned} \ell = (2\alpha _{11} - 5)(\lambda +2\mu )/2(1-\nu )-(2m-\nu \lambda )/(1-2\nu ) \end{aligned}$$

59 $$\begin{aligned} m = \Bigl ( (\alpha _{11} - \alpha _{22})/2( 1 +\nu ) - 1\Bigr )(\lambda + 2\mu ) -\frac{\mu }{2} \end{aligned}$$

60 $$\begin{aligned} n = 2(\lambda + m -2\nu (\lambda + 3\mu +m) -2\mu \alpha _{23}) \end{aligned}$$

## Engineering application of the third order elastic constants

When the third-order elastic constants are a priori obtained for the structural materials, the stress situation in various sites in the material can be estimated by measuring the elastic wave propagation times. These applications are given in both Japanese and USA patents (Takahashi 2007, 2012).

**Figure 2:**
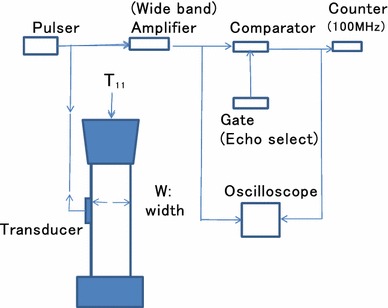
Schematic diagram of stress measurement system.

Figure 2 shows the stress measurement for structural object under an applied load of $T_{11}$ using an elastic wave. We denoted the width of the non-loaded object by $W$.

The propagation time $t$ is defined by the following,

61 $$\begin{aligned} t = \frac{ W (1-\nu T_{11}/E )}{ V_0 (1 + \alpha _{22} T_{11}/E)} \end{aligned}$$

where $V_0$ is the propagation velocity of the elastic wave in the non-loaded object and $\alpha _{22}$ the strain dependency coefficient.

Formula (61) is rearranged to give

62 $$\begin{aligned} T_{11} = -\frac{E}{\alpha _{22} + \nu } \Bigl (\frac{ \Delta t }{t_0} \Bigl ), \quad \Delta t =t - t_0 \end{aligned}$$

where $t_0$ is propagation time in the non-loaded object and ${\Delta t}/{t_0}$ is the change ratio in propagation time with $t_0=W/V_0$.

If we know $\alpha _{22}$, E, and $\nu$, the value of $T_{11}$ can be obtained through measuring ${\Delta t}/{t_0}$ using formula (62).

**Figure 3:**
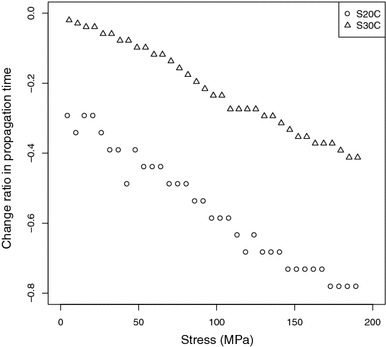
Change ratio in propagation time vs stresses.

Figure 3 shows the relationship between the change ratio in propagation time with stress for the carbon steel samples S20C and S30C.

**Table 1:** Chemical composition of S20C and S30C specimens (wt%)

Sample	C	Si	Mn
S20C	0.22	0.29	0.52
S30C	0.32	0.31	0.81

Table 1 shows the chemical composition for both S20C and S30C samples of which were used in the stress experiment.

## Discussion

The coordinates used for describing an isotropic solid before deformation, the large non-linear displacement, and the infinitesimal displacement of the elastic wave have been clearly defined using orthogonal Cartesian coordinates. The expressions for stress and strain as presented in Eqs. (6) and (21) are polynomial and the strain tensor is symmetric, so their development becomes comparatively easy. The theory reported by Hughes and Kelly (1953) for the measurement of the third-order elastic constants of practical materials is difficult because of its unique treatment of the strain and the use of special coordinate functions as well as of the bulk modulus to obtain the third-order elastic constants. Our method using the tensile testing machine is easy to apply in the measurement of the parameters necessary for the determination of these elastic constants. Murnaghans third-order elastic constants $\ell, m, n$ can be obtained from their changes with time. We demonstrated that they are very useful for the evaluation of stress in structural materials and in the identification of materials. The density of the material is not required in obtaining these changes and hence the method is of great benefit in practical applications. In establishing the equation of motion of elastic wave, the infinitesimal strain, stress, and Jacobian are found to be the main elements along with the load stress. The carbon content and the stress applied to the carbon steel are varied systematically in accordance with the relation of frequency ratio with stress (Takahashi et al. 1978) and also from the relation of stress with the time rate of change of the longitudinal and transverse waves (Takahashi and Motegi 1987). These provide the fundamental data for stress measurements and are essential for the identification of materials. In stress measurements of actual structures, it is necessary to collect quantitative data under various conditions as laboratory conditions are hard to establish.

## Conclusion

The change of propagation velocity of elastic wave due to the static stress cannot be expressed essentially by the infinitesimal elastic theory. The second and the third-order elastic moduli and additional elastic strains contribute to the change in propagation velocity. The analysis of the applied stress, strain, infinitesimal stress of elastic wave and its strain related to an isotropic elastic body were performed using the theory of Murnaghan combined with the Lagrangian description. The formulas are lengthy, but the contents is simple. Our analytical procedure is different to that of Hughes and Kelly (1953), however the results obtained were equivalent in regard to the equations giving the propagation velocity of an elastic wave.

Finally this paper demonstrated that the third-order elastic constants for engineering materials were useful in estimating the unknown stresses of structural materials.

## Nomenclature

$$a_1$$, $$a_2$$, $$a_3$$
coordinate of the non-deformed state
$$X_1$$, $$X_2$$, $$X_3$$
coordinate of the statically and finitely deformed state
$$x_1$$, $$x_2$$, $$x_3$$
coordinate of the state where dynamic and infinitesimal deformations are superposed on the finitely deformed state.
$$U_1$$, $$U_2$$, $$U_3$$
coordinated for finite displacement
$$u_1$$, $$u_2$$, $$u_3$$
coordinates for infinitesimal displacement
$$\eta _{ij}$$
static strain coefficients in the finitely deformed state
$$\hat{\eta }_{ij}$$
infinitesimal strain coefficients of the elastic wave
$$\bar{\eta }_{ij}$$
coefficients for total strain superposed by the infinitesimal strain of $\hat{\eta }_{ij}$ on the static strain
$$\delta _{ij}$$
Kronecker’s delta function
$$I_1$$, $$I_2$$, $$I_3$$
strain invariants
$$\rho _0$$
density in the non-deformed state
$$\rho _0\phi$$
free energy per unit volume
$$J_{ij}$$
Jacobian matrix in the finitely deformed state
$$\hat{J}$$, $$\bar{J}$$
Jacobian matrix in the infinitesimal deformed state, and the total Jacobian matrix
$$l$$, $$m$$, $$n$$
Murnahan’s third-order elastic constants
$$\lambda$$, $$\mu$$
Lamé constants
E: Young’s modulus
$$T_{ij}$$
stress in the finitely deformed state
$$\hat{T}_{ij}$$
stress in the infinitesimal deformed state
$$\bar{T}_{ij}$$
total stress superposed by the $\hat{T}_{ij}$ on the stress $T_{ij}$
$$\omega$$, $$\kappa$$
angular frequency and wave number
$$\nu$$
Poisson’s ratio
$$A$$
amplitude
$$V_{ij}$$
elastic wave velocity
$$\alpha$$, $$\beta$$
greek letter subscripts indicate summation over all spacial indices 1, 2 and 3, for example $\eta _{1\alpha }= \eta _{11} + \eta _{12} + \eta _{13}$

## Appendix A

The following presents a calculation of $\hat{\eta }_{11}$ using formula (6) of the main text

63 $$\begin{aligned} \hat{\eta }_{11} = \left( \left( \delta _{\alpha 1}+\frac{\partial U_\alpha }{\partial a_1}\right) \left( \delta _{\beta 1} + \frac{\partial U_\beta }{\partial a_1}\right) \right) \left( \frac{\partial u_\alpha }{\partial X_\beta }\right) \end{aligned}$$

64 $$\begin{aligned} \hat{\eta }_{11} &=\Bigl (1+\frac{\partial U_1}{\partial a_1}\Bigr )^2 \dot{\eta _{11}} + \Bigl (1+\frac{\partial U_1}{\partial a_1}\Bigr )\frac{\partial U_2}{\partial a_1} \dot{\eta _{12}}+ \Bigl (1+\frac{\partial U_1}{\partial a_1}\Bigr )\frac{\partial U_3}{\partial a_1} \dot{\eta _{13}} + \frac{\partial U_2}{\partial a_1} \Bigl (1+\frac{\partial U_1}{\partial a_1}\Bigr ) \dot{\eta _{21}} \nonumber \\&\quad +\frac{\partial U_2}{\partial a_1}\frac{\partial U_2}{\partial a_1} \dot{\eta _{22}} +\frac{\partial U_2}{\partial a_1}\frac{\partial U_3}{\partial a_1} \dot{\eta _{23}} \nonumber \\&\quad + \frac{\partial U_3}{\partial a_1} \Bigl (1+\frac{\partial U_1}{\partial a_1}\Bigl ) \dot{\eta _{31}} + \frac{\partial U_3}{\partial a_1}\frac{\partial U_2}{\partial a_1} \dot{\eta _{32}} +\frac{\partial U_3}{\partial a_1}\frac{\partial U_3}{\partial a_1} \dot{\eta _{33}} \end{aligned}$$

We then neglect higher-order terms of $\frac{\partial U_2}{\partial a_1}\frac{\partial U_2}{\partial a_1}$ and set $\frac{\partial u_1}{\partial X_1}=\dot{\eta _{11}}$, $\frac{\partial u_1}{\partial X_2}+\frac{\partial u_2}{\partial X_1}=\dot{\eta }_6$,

$$\frac{\partial u_1}{\partial X_3}+\frac{\partial u_3}{\partial X_1}=\dot{\eta }_5$$, $$\frac{\partial u_2}{\partial X_3}+\frac{\partial u_3}{\partial X_2}=\dot{\eta _4}$$

With the change in subscripts of $\dot{\eta }$ given as follows,

$$\dot{\eta _{11}} \rightarrow \dot{\eta _1}$$, $$\dot{\eta _{22}} \rightarrow \dot{\eta _2}$$, $$\dot{\eta _{33}} \rightarrow \dot{\eta _3}$$, $$\dot{\eta _{32}} \rightarrow \dot{\eta _4}$$, $$\dot{\eta _{31}} \rightarrow \dot{\eta _5}$$, $$\dot{\eta _{21}} \rightarrow \dot{\eta _6}$$

we obtain

65 $$\begin{aligned} \hat{\eta }_{11} \approx \Bigl ( 1+2\frac{\partial U_1}{\partial a_1}\Bigl )\dot{\eta _1} + \frac{\partial U_2}{\partial a_1}\dot{\eta _6} + \frac{\partial U_3}{\partial a_1}\dot{\eta _5} \end{aligned}$$

Also changing the subscripts as prescribed by $1 \rightarrow 2,\, 2 \rightarrow 3,\, 3 \rightarrow 1$ and $4 \rightarrow 5,\, 5 \rightarrow 6,\, 6 \rightarrow 4$ produces the other coefficients of infinitesimal strain,

66 $$\begin{aligned} \hat{\eta }_{22} \approx \Bigl ( 1+2\frac{\partial U_2}{\partial a_2}\Bigl )\dot{\eta _2} + \frac{\partial U_3}{\partial a_2}\dot{\eta _4} + \frac{\partial U_1}{\partial a_2}\dot{\eta _6} \end{aligned}$$

67 $$\begin{aligned} \hat{\eta }_{33} \approx \Bigl ( 1+2\frac{\partial U_3}{\partial a_3}\Bigl )\dot{\eta _3} + \frac{\partial U_1}{\partial a_3}\dot{\eta _5} + \frac{\partial U_2}{\partial a_3}\dot{\eta _4} \end{aligned}$$

68 $$\begin{aligned} \hat{\eta }_{23}=\frac{1}{2}\left( \left( \delta _{\alpha 2}+\frac{\partial U_\alpha }{\partial a_2}\right) \left( \delta _{\beta 3}+\frac{\partial U_\beta }{\partial a_3}\right) \right) \left( \frac{\partial u_\alpha }{\partial X_\beta }+\frac{\partial u_\beta }{\partial X_\alpha }\right) \end{aligned}$$

In similar manner with $\hat{\eta }_{11}$, replacing $\alpha$ and $\beta$ with 1, 2, 3, and summing up each term, we find on neglecting higher orders involving the derivative terms,

69 $$\begin{aligned} \hat{\eta }_{23}=\frac{\partial U_2}{\partial a_3}\dot{\eta }_2+\frac{\partial U_3}{\partial a_2}\dot{\eta }_3+\frac{1}{2}\frac{\partial U_1}{\partial a_3}\dot{\eta }_6+\frac{1}{2}\frac{\partial U_1}{\partial a_2}\dot{\eta }_5 +\frac{1}{2}\left( 1+\frac{\partial U_2}{\partial a_2}+\frac{\partial U_3}{\partial a_3}\right) \dot{\eta }_4 \end{aligned}$$

70 $$\begin{aligned} \hat{\eta }_{31} = \frac{\partial U_3}{\partial a_1}\dot{\eta _3} + \frac{\partial U_1}{\partial a_3}\dot{\eta _1}+ \frac{1}{2}\frac{\partial U_2}{\partial a_1}\dot{\eta _4}+ \frac{1}{2}\frac{\partial U_2}{\partial a_3}\dot{\eta _6}+\frac{1}{2}\Bigl (1+\frac{\partial U_3}{\partial a_3} +\frac{\partial U_1}{\partial a_1}\Bigl ) \dot{\eta _5} \end{aligned}$$

71 $$\begin{aligned} \hat{\eta }_{12} = \frac{\partial U_1}{\partial a_2}\dot{\eta _1} + \frac{\partial U_2}{\partial a_1}\dot{\eta _2}+ \frac{1}{2}\frac{\partial U_3}{\partial a_2}\dot{\eta _5}+ \frac{1}{2}\frac{\partial U_3}{\partial a_1}\dot{\eta _4}+\frac{1}{2}\Bigl (1+\frac{\partial U_1}{\partial a_1} +\frac{\partial U_2}{\partial a_2}\Bigl ) \dot{\eta _6} \end{aligned}$$

We then use the following expressions

72 $$\begin{aligned} \frac{\partial U_1}{\partial a_1} = \eta _1,\, \frac{\partial U_2}{\partial a_2} = \eta _2,\, \frac{\partial U_3}{\partial a_3} = \eta _3,\, \frac{\partial U_3}{\partial a_2} = \eta _4,\, \frac{\partial U_3}{\partial a_1} = \eta _5,\, \frac{\partial U_2}{\partial a_1} = \eta _6 \end{aligned}$$

and the following relation

73 $$\begin{aligned} \frac{\partial U_\alpha }{\partial a_\beta } = \frac{\partial U_\beta }{\partial a_\alpha } \end{aligned}$$

which can be applied in the case of an isotropic object, to simplify the expression for each of the infinitesimal strains.

## Appendix B

74 $$\begin{aligned} \frac{\partial (\rho _0 \phi )}{\partial \eta _{22}}&= (\lambda +2\mu )I_1+(\ell +2m)I^2_1-2m I_2-2(\mu +m I_1)I_1 \nonumber \\&\quad +2\eta _{22}(\mu +m I_1)+n(\eta _{33}\eta _{11}-\eta _{31}\eta _{13}) \end{aligned}$$

75 $$\begin{aligned} \frac{\partial (\rho _0 \phi )}{\partial \eta _{33}}=\lambda I_1+\ell I^2_1-2m I_2 +2\eta _{33}(\mu +m I_1)+n(\eta _{11}\eta _{22}-\eta _{12}\eta _{21}) \end{aligned}$$

76 $$\begin{aligned} \frac{\partial (\rho _0 \phi )}{\partial \eta _{23}}= 2(\mu +m I_1)\eta _{32}+n(\eta _{12}\eta _{31}-\eta _{11}\eta _{32}) \end{aligned}$$

77 $$\begin{aligned} \frac{\partial (\rho _0 \phi )}{\partial \eta _{31}}= 2(\mu +m I_1)\eta _{13}+n(\eta _{23}\eta _{12}-\eta _{22}\eta _{13}) \end{aligned}$$

78 $$\begin{aligned} \frac{\partial (\rho _0 \phi )}{\partial \eta _{12} } &= 2(\mu +m I_1)\eta _{21}+n(\eta _{31}\eta _{23}-\eta _{33}\eta _{21}) \end{aligned}$$

Derivatives of the free energy with respect to $\eta _{32}$, $\eta _{13}$, and $\eta _{21}$ are equivalent to those above because of the symmetry $\eta _{ij}=\eta _{ji}$.

Given the definition of the invariants $\hat{I}_1$ and $\hat{I}_2$ in terms of the infinitesimal strain,

79 $$\begin{aligned} \hat{I}_1=\hat{\eta }_{11}+\hat{\eta }_{22}+\hat{\eta }_{33} \end{aligned}$$

and

80 $$\begin{aligned} \hat{I}_2 &=(I_1 - \eta _{11} )\hat{\eta }_{11} +(I_1 -\eta _{22})\hat{\eta }_{22} +(I_1-\eta _{33})\hat{\eta }_{33} \nonumber \\&\quad -\eta _{32} \hat{\eta }_{23} - \eta _{23}\hat{\eta }_{32}-\eta _{13} \hat{\eta }_{31}- \eta _{31} \hat{\eta }_{13}-\eta _{21} \hat{\eta }_{12} -\eta _{12}\hat{\eta }_{21} \nonumber \\ &= I_1\hat{I}_1-\eta _{11}\hat{\eta }_{11}-\eta _{22}\hat{\eta }_{22}-\eta _{33}\hat{\eta }_{33}-\eta _{32}\hat{\eta }_{23} \nonumber \\&\quad -\eta _{23}\hat{\eta }_{32}-\eta _{13}\hat{\eta }_{31}-\eta _{31}\hat{\eta }_{13}-\eta _{21}\hat{\eta }_{12} -\eta _{12}\hat{\eta }_{21} \end{aligned}$$

then their partial derivatives follow directly;

$$\frac{\partial I_1}{\partial \eta _{11}}=1$$, $$\frac{\partial I_1}{\partial \eta _{23}}=0$$,    $$\frac{\partial I_2}{\partial \eta _{11}}=\eta _{22}+\eta _{33}$$, $$\frac{\partial I_2}{\partial \eta _{23}}=-\eta _{32}$$,

$$\frac{\partial I_3}{\partial \eta _{11}}=\eta _{22}\eta _{33}-\eta _{23}\eta _{32}$$,    $$\frac{\partial I_3}{\partial \eta _{23}}=\eta _{12}\eta _{31}-\eta _{11}\eta _{32}$$.

The expression of the first invariant can be used to obtain

81 $$\begin{aligned} \frac{\partial I_2}{\partial \eta _{11}}= I_1 - \eta _{11} \end{aligned}$$

## Appendix C

From the definition of the Jacobian matrix, we have the following expressions:

82 $$\begin{aligned} \hat{J}_{11}=\frac{\partial u_1}{\partial a_1}=\left( 1+\frac{\partial U_1}{\partial a_1}\right) \frac{\partial u_1}{\partial X_1} + \frac{\partial U_2}{\partial a_1}\frac{\partial u_1}{\partial X_2} + \frac{\partial U_3}{\partial a_1}\frac{\partial u_1}{\partial X_3} \end{aligned}$$

83 $$\begin{aligned} \hat{J}_{12}=\frac{\partial u_1}{\partial a_2}=\frac{\partial U_1}{\partial a_2}\frac{\partial u_1}{\partial X_1} + \left( 1+\frac{\partial U_2}{\partial a_2}\right) \frac{\partial u_1}{\partial X_2} + \frac{\partial U_3}{\partial a_2}\frac{\partial u_1}{\partial X_3} \end{aligned}$$

84 $$\begin{aligned} \hat{J}_{13}&= \frac{\partial u_1}{\partial a_3}=\frac{\partial U_1}{\partial a_3}\frac{\partial u_1}{\partial X_1} + \frac{\partial U_2}{\partial a_3}\frac{\partial u_1}{\partial X_2} + \left( 1+\frac{\partial U_3}{\partial a_3}\right) \frac{\partial u_1}{\partial X_3} \end{aligned}$$

Similar expression for $\hat{J}_{21}$, $\hat{J}_{22}$, and the others can be obtained from the formula,

$$\frac{\partial u_i}{\partial a_j}$$ =$$( \delta _{j \alpha }+\frac{\partial U_\alpha }{\partial a_j})\frac{\partial u_i}{\partial X_\alpha }$$.

## Appendix D

We develop here an approximation for the partial derivative of $\hat{\phi }$ with respect to static strain $\eta _{11}$ to illustrate our procedure:

85 $$\begin{aligned} \frac{\partial \hat{\phi }}{\partial \eta _{11}}&= \lambda \bar{I}_1+ \ell \bar{I}_1 ^2 -2m \bar{I}_2 +2\bar{\eta }_{11}(\mu + m \bar{I}_1) +n(\bar{\eta }_{22}\bar{\eta }_{33} \nonumber \\&\quad -\bar{\eta }_{23}\bar{\eta }_{32})- \lambda I_1- \ell I_1^2 + 2m I_2 - 2\eta _{11}(\mu + m I_1) -n(\eta _{22} \eta _{33}- \eta _{23} \eta _{32}) \end{aligned}$$

86 $$\begin{aligned} &=\lambda \hat{I}_1+ \ell ( \bar{I}_1 - I_1)(\bar{I}_1 + I_1)-2m \hat{I}_2 +2\mu \hat{\eta }_{11} + 2m(\bar{\eta }_{11} \bar{I}_1-\eta _{11} I_1) \nonumber \\& \quad+ n(\bar{\eta }_{22}\bar{\eta }_{33}-\eta _{22}\eta _{33}-\bar{\eta }_{23}\bar{\eta }_{32}+\eta _{23}\eta _{32}) \end{aligned}$$

87 $$\begin{aligned} &=(\lambda + \ell ( 2 I_1 +\hat{I}_1))\hat{I}_1 -2m \hat{I}_2+2\mu \hat{\eta }_{11} + 2m(\bar{\eta }_{11} I_1 + \eta _{11}\hat{I}_1+ \hat{\eta }_{11} \hat{I}_1) \nonumber \\&\quad+ n(\hat{\eta }_{22} \eta _{33}+\eta _{22} \hat{\eta }_{33} + \hat{\eta }_{22} \hat{\eta }_{33}-\hat{\eta }_{23}\eta _{32}- \eta _{23}\hat{\eta }_{32}-\hat{\eta }_{23} \hat{\eta }_{32}) \end{aligned}$$

88 $$\begin{aligned} &\approx(\lambda +2\ell I_1) \hat{I}_1 \nonumber \\& \quad -2m(I_1 \hat{I}_1-\eta _{11} \hat{\eta }_{11} -\eta _{22} \hat{\eta }_{22} - \eta _{33} \hat{\eta }_{33}- \eta _{32} \hat{\eta }_{23} \nonumber \\& \quad- \eta _{23} \hat{\eta }_{32} -\eta _{13} \hat{\eta }_{31}-\eta _{31} \hat{\eta }_{13} - \eta _{21} \hat{\eta }_{12}- \eta _{12} \hat{\eta }_{21}) \nonumber \\& \quad +2\mu \hat{\eta }_{11} + 2m I_1 \hat{\eta }_{11} + 2m \eta _{11} \hat{I}_1 + n ( \eta _{33} \hat{\eta }_{22} + \eta _{22} \hat{\eta }_{33} \nonumber \\& \quad - \eta _{32} \hat{\eta }_{23} - \eta _{23}\hat{\eta }_{32} ) \end{aligned}$$

89 $$\begin{aligned} &=(\lambda +2\ell I_1- 2m I_1+2m \eta _{11}) \hat{I}_1 +(2m \eta _{11}+ 2\mu +2m I_1)\hat{\eta }_{11} \nonumber \\& \quad + (2m \eta _{22}+n \eta _{33})\hat{\eta }_{22} +(2m \eta _{33}+n \eta _{22}) \hat{\eta }_{33} +( 2m-n)(\eta _{32} \hat{\eta }_{23} \nonumber \\& \quad + \eta _{32} \hat{\eta }_{23} +\eta _{23} \hat{\eta }_{32})+ 2m (\eta _{21} \hat{\eta }_{12}+\eta _{12} \hat{\eta }_{21}+\eta _{13} \hat{\eta }_{31}+\eta _{31} \hat{\eta }_{13}) \end{aligned}$$

90 $$\begin{aligned} &=(\lambda + 2\ell I_1+2m \eta _{11}+ 2m \eta _{11} \nonumber \\& \quad + 2\mu )\hat{\eta }_{11} +( \lambda +2(\ell -m) I_1 +2m \eta _{11}+2m\eta _{22}+ n\eta _{33})\dot{\eta }_{22} \nonumber \\& \quad + (\lambda + 2(\ell -m)I_1 + 2m\eta _{11} +2m \eta _{33} +n \eta _{22}) \hat{\eta }_{33} \nonumber \\& \quad + 2m (\eta _{12}\hat{\eta }_{21} + \eta _{21}\hat{\eta }_{12} + \eta _{13}\hat{\eta }_{31} + \eta _{31} \hat{\eta }_{13} ) \nonumber \\& \quad -( 2m-n)(\eta _{23} \hat{\eta }_{32} + \eta _{32} \hat{\eta }_{23}) \end{aligned}$$

91 $$\begin{aligned} &=(\lambda + 2\mu + 2\ell I_1 +4m \eta _{11})\hat{\eta }_{11} +( \lambda +2\ell I_1- (2m -n) \eta _{33})\hat{\eta }_{22}+ (\lambda + 2\ell I_1 \nonumber \\& \quad - ( 2m-n)\eta _{22}) \hat{\eta }_{33} + (2m -n)(\eta _{23} \hat{\eta }_{32} + \eta _{32}\hat{\eta }_{23}) \nonumber \\& \quad + 2m(\eta _{13}\hat{\eta }_{31} +\eta _{31}\hat{\eta }_{13} + \eta _{12}\hat{\eta }_{21} + \eta _{21}\hat{\eta }_{12}) \end{aligned}$$

## Appendix E

We develop here an approximation for the stress in the infinitesimal deformed state using $\hat{T}_{11}$ to illustrate our procedure:

92 $$\begin{aligned} \hat{T}_{11} &=\hat{J}_{1 \alpha }\frac{\partial \phi }{\partial \eta _{\alpha 1}} + J_{1\alpha }\frac{\partial \hat{\phi }}{\partial \eta _{\alpha 1}} \nonumber \\ &=\frac{\partial u_1}{\partial a_2}( \lambda I_1 + \ell I_1^2 - 2m I_2 +2\eta _{11} ( \mu +m I_1) +n (\eta _{22}\eta _{33}- \eta _{23}\eta _{32} )) \nonumber \\&\quad + \frac{\partial u_1}{\partial a_2}(2(\mu + m I_1)\eta _{12} + n(\eta _{32} \eta _{13} - \eta _{33} \eta _{12})) \nonumber \\&\quad +\frac{\partial u_1}{\partial a_3}(2(\mu + m I_1)\eta _{13} + n(\eta _{23} \eta _{12} - \eta _{22} \eta _{1 3})) \nonumber \\&\quad + \left( 1+\frac{\partial U_1}{\partial a_1}\right) (( \lambda +2\mu +2\ell I_1 +4m \eta _{11})\hat{\eta }_{11} +(\lambda +2\ell I_1 \nonumber \\&\quad -(2m-n)\eta _{33})\hat{\eta }_{22} \nonumber \\&\quad + (\lambda + 2\ell I_1 - (2m-n) \eta _{22})\hat{\eta }_{33} + (2m - n)(\eta _{23}\hat{\eta }_{32}- \eta _{32}\hat{\eta }_{23}) \nonumber \\&\quad +2m(\eta _{13}\hat{\eta }_{31} + \eta _{31}\hat{\eta }_{13} + \eta _{12}\hat{\eta }_{21} +\eta _{21}\hat{\eta }_{12})) \nonumber \\&\quad + \frac{\partial U_1}{\partial a_2}(( 2m -n)\eta _{12}\hat{\eta }_{33} + 2m\eta _{12}( \hat{\eta }_{11} + \hat{\eta }_{22)} \nonumber \\& \quad + (2\mu +2m I_1 - n\eta _{33})\hat{\eta }_{12} + n(\eta _{32} \hat{\eta }_{13} - \eta _{13} \hat{\eta }_{32})) \nonumber \\&\quad + \frac{\partial U_1}{\partial a_3}((2m-n)\eta _{13}\hat{\eta }_{22} + 2m\eta _{13}( \hat{\eta }_{33} + \hat{\eta }_{11}) \nonumber \\&\quad + (2\mu +2m I_1 -n \eta _{22} ) \hat{\eta }_{13} +n( \eta _{23}\hat{\eta }_{12} + \eta _{12}\hat{\eta }_{23})) \end{aligned}$$

93 $$\begin{aligned} &\approx \frac{\partial u_1}{\partial a_1}( \lambda I_1 + 2\mu \eta _{11}) +2\mu \eta _{12} \frac{\partial u_1}{\partial a_2} \nonumber \\& \quad + 2\mu \eta _{13} \frac{\partial u_1}{\partial a_3} + \frac{\partial U_1}{\partial a_1}(( \lambda +2\mu )\hat{\eta }_{11} +\lambda (\hat{\eta }_{22} + \hat{\eta }_{33})) \nonumber \\& \quad + (\lambda +2\mu +2\ell I_1+4m\eta _{11})\hat{\eta }_{11}+(\lambda +2\ell I_1)(\hat{\eta }_{22}+\hat{\eta }_{33}) \nonumber \\& \quad + (2m-n)(\eta _{23}\hat{\eta }_{32} + \eta _{32} \hat{\eta }_{23} - \eta _{33}\hat{\eta }_{22} - \eta _{22}\hat{\eta }_{33}) \nonumber \\& \quad +2m(\eta _{21} \hat{\eta }_{12}+\eta _{12} \hat{\eta }_{21}+ \eta _{13} \hat{\eta }_{31} + \eta _{31} \hat{\eta }_{13}) \nonumber \\& \quad + 2\mu \frac{\partial U_1}{\partial a_2} \hat{\eta }_{12}+ 2\mu \frac{\partial U_1}{\partial a_3} \hat{\eta }_{13} \end{aligned}$$

Using the formulas for ${\partial u_1}/{\partial a_1}$ etc in Appendix C, we then obtain

94 $$\begin{aligned} \hat{T}_{11} &\approx (\lambda I_1 + 2 \mu \eta _{11})\left(\left( 1+\frac{\partial U_1}{\partial a_1}\right) \frac{\partial u_1}{\partial X_1} + \frac{\partial U_2}{\partial a_1}\frac{\partial u_1}{\partial X_2} +\frac{\partial U_3}{\partial a_1}\frac{\partial u_1}{\partial X_3} \right)\nonumber \\&\quad +2\mu \eta _{12}\left(\frac{\partial U_1}{\partial a_2}\frac{\partial u_1}{\partial X_1}+ \left( 1+\frac{\partial U_2}{\partial a_2}\right) \frac{\partial u_1}{\partial X_2}+ \frac{\partial U_3}{\partial a_2} \frac{\partial u_1}{\partial X_3}\right) \nonumber \\&\quad +2\mu \eta _{13} \left(\frac{\partial U_1}{\partial a_3}\frac{\partial u_1}{\partial X_1}+ \frac{\partial U_2}{\partial a_3}\frac{\partial u_1}{\partial X_2} + \left( 1+\frac{\partial U_3}{\partial a_3}\right) \frac{\partial u_1}{\partial X_3}\right) \nonumber \\&\quad + \frac{\partial U_1}{\partial a_1}(\lambda \hat{I}_1 +2\mu \hat{\eta }_{11}) +(\lambda +2\ell I_1) \hat{I}_1 + ( 2\mu +4m \eta _{11}) \hat{\eta }_{11} \nonumber \\&\quad +(2m-n)(\eta _{32}\hat{\eta }_{23}+\eta _{23}\hat{\eta }_{32}- \eta _{33}\hat{\eta }_{22}- \eta _{22}\hat{\eta }_{33}) \nonumber \\&\quad +2m( \eta _{31} \hat{\eta }_{13}+\eta _{13} \hat{\eta }_{31}+\eta _{12} \hat{\eta }_{21}+\eta _{21} \hat{\eta }_{12}) + 2\mu \frac{\partial U_1}{\partial a_2} \hat{\eta }_{12}+2\mu \frac{\partial U_1}{\partial a_3} \hat{\eta }_{13} \end{aligned}$$

95 $$\begin{aligned} &\approx (\lambda I_1 +2\mu \eta _{11})\frac{\partial u_1}{\partial X_1}+ 2\mu \frac{\partial u_1}{\partial X_2} + 2\mu \eta _{13}\frac{\partial u_1}{\partial X_3} \nonumber \\&\quad + \left( \lambda +2 \ell I_1 + \lambda \frac{\partial U_1}{\partial a_1}\right) \hat{I}_1+\left( 2\mu \frac{\partial U_1}{\partial a_1} +2\mu + 4m \eta _{11}\right) \hat{\eta }_{11} \nonumber \\&\quad + (2m-n) (\eta _{32} \hat{\eta }_{23}+ \eta _{23} \hat{\eta }_{32}- \eta _{33} \hat{\eta }_{22} - \eta _{22} \hat{\eta }_{33}) \nonumber \\&\quad + 2m( \eta _{31} \hat{\eta }_{13} + \eta _{13} \hat{\eta }_{31} + \eta _{12} \hat{\eta }_{21} + \eta _{21} \hat{\eta }_{12}) + 2\mu \Bigl ( \frac{\partial U_1}{\partial a_2} \hat{\eta }_{12} + \frac{\partial U_1}{\partial a_3} \hat{\eta }_{13}\Bigl ) \end{aligned}$$

96 $$\begin{aligned} & \approx ( \lambda I_1 +2\mu \eta _{11}) \frac{\partial u_1}{\partial X_1} + 2 \mu \eta _{12} \frac{\partial u_1}{\partial X_2} + 2\mu \eta _{13} \frac{\partial u_1}{\partial X_3} + \left( \lambda + 2\ell I_1 + \lambda \frac{\partial U_1}{\partial a_1} \right) \nonumber \\&\quad \Biggl(\left( 1+2\frac{\partial U_1}{\partial a_1}\right) \dot{\eta _1} + \left( 1+2\frac{\partial U_2}{\partial a_2}\right) \dot{\eta _2}+ \left( 1+2\frac{\partial U_3}{\partial a_3}\right) \dot{\eta _3} \nonumber \\&\quad + \left( \frac{\partial U_3}{\partial a_2} + \frac{\partial U_2}{\partial a_3}\right) \dot{\eta _4} +\left( \frac{\partial U_1}{\partial a_3} + \frac{\partial U_3}{\partial a_1}\right) \dot{\eta _5} +\left( \frac{\partial U_2}{\partial a_1} + \frac{\partial U_1}{\partial a_2}\right) \dot{\eta _6}\Biggl) \nonumber \\&\quad + \left(2\mu \left( 1+\frac{\partial U_1}{\partial a_1}\right) + 4m \eta _{11}\right) \left(\left( 1 + 2\frac{\partial U_1}{\partial a_1}\right) \dot{\eta _1} + \frac{\partial U_2}{\partial a_1}\dot{\eta _6} + \frac{\partial U_3}{\partial a_1}\dot{\eta _5}\right) \nonumber \\&\quad +(2m-n) \left( \eta _{32} \frac{1}{2} \dot{\eta _4} + \eta _{23} \frac{1}{2} \dot{\eta _4}-\eta _{33}\dot{\eta _2} - \eta _{22}\dot{\eta _3}\right) \nonumber \\&\quad + 2m \left( \eta _{31}\frac{1}{2}\dot{\eta _5} + \eta _{13}\frac{1}{2}\dot{\eta _5} + \eta _{12}\frac{1}{2}\dot{\eta _6} + \eta _{21}\frac{1}{2}\dot{\eta _6} \right) + 2\mu \frac{\partial U_1}{\partial a_2} \frac{1}{2}\dot{\eta _6} + 2\mu \frac{\partial U_1}{\partial a_3} \frac{1}{2}\dot{\eta _5} \end{aligned}$$

97 $$\begin{aligned} \hat{T}_{11} &\approx ( \lambda I_1 +2\mu \eta _{11}) \dot{\eta _1} +2\mu \eta _{12}\frac{\partial u_1}{\partial X_2} + 2\mu \eta _{13}\frac{\partial u_1}{\partial X_3} \nonumber \\&\quad + \left(\lambda (1 +2\eta _1) \dot{\eta _1} + ( 1+ 2\eta _2) \dot{\eta _2} + ( 1+ 2\eta _3) \dot{\eta _3} + \eta _4 \dot{\eta _4} + \eta _5 \dot{\eta _5} + \eta _6 \dot{\eta _6}\right) \nonumber \\&\quad + ( 2\ell I_1 + \lambda \eta _1 )( \dot{\eta _1} + \dot{\eta _2} + \dot{\eta _3}) +2\mu \left(( 1+3\eta _1) \dot{\eta _1} +\frac{\partial U_2}{\partial a_1}\dot{\eta _6} + \frac{\partial U_3}{\partial a_1}\dot{\eta _5}\right) \nonumber \\&\quad +4m \eta _1 \dot{\eta _1} + ( 2m -n)\left( \frac{1}{2}(\eta _{23} + \eta _{32}) \dot{\eta _4} - \eta _3 \dot{\eta _2} - \eta _2 \dot{\eta _3} \right) \nonumber \\&\quad +2m \left( \frac{1}{2}(\eta _{31} + \eta _{13})\dot{\eta _5} + \frac{1}{2} (\eta _{12} +\eta _{21})\dot{\eta _6}\right) + \mu \frac{\partial U_1}{\partial a_2} \dot{\eta _6} + \frac{\partial U_1}{\partial a_3} \dot{\eta _5} \end{aligned}$$

98 $$\begin{aligned} \hat{T}_{11} &\approx ( \lambda I_1 + 2\mu \eta _1 + \lambda ( 1+ 2\eta _1) +2 \ell I_1 + \lambda \eta _1 \nonumber \\&\quad + 2\mu ( 1+ 3\eta _1) +4m\eta _1) \dot{\eta _1} + (\lambda (1+2\eta _2) + \lambda \eta _1 +2\ell I_1 -(2m-n)\eta _3)\dot{\eta _2} \nonumber \\&\quad + (\lambda (1+2\eta _3) + \lambda \eta _1 +2\ell I_1 -(2m-n)\eta _2)\dot{\eta _3} + \left( \lambda \eta _4 + \frac{(2m-n)}{2} \eta _4\right) \dot{\eta _4} \nonumber \\&\quad +\left( \lambda \eta _5 + 2\mu \frac{\partial U_3}{\partial a_1} +m \eta _5 + \mu \frac{\partial U_1}{\partial a_3} \right) \dot{\eta _5} \nonumber \\&\quad +\left( \lambda \eta _6 + 2\mu \frac{\partial U_2}{\partial a_1} +m \eta _6 + \mu \frac{\partial U_1}{\partial a_2} \right) \dot{\eta _6} + 2\mu \left( \eta _{12} \frac{\partial u_1}{\partial X_2} + \eta _{13} \frac{\partial u_1}{\partial X_3} \right) \end{aligned}$$

99 $$\begin{aligned} \hat{T}_{11} &=( \lambda + 2\mu +( \lambda + 2\ell ) I_1 + ( 3\lambda +8\mu + 4m) \eta _1) \dot{\eta _1} \nonumber \\&\quad +( \lambda + ( \lambda +2\ell ) I_1 + \lambda \eta _2 - ( \lambda + 2m - n) \eta _3) \dot{\eta _2} \nonumber \\&\quad + (\lambda +( \lambda + 2\ell ) I_1 - ( \lambda + 2m -n) \eta _2 + \lambda \eta _3) \dot{\eta _3} +\frac{1}{2}(\lambda + 2m-n)\eta _4 \dot{\eta _4} \nonumber \\&\quad + \frac{1}{2} (2\lambda +3\mu +2m)(\eta _5 \dot{\eta _5} + \eta _6 \dot{\eta _6}) + 2\mu \Bigl ( \eta _{12}\frac{\partial u_1}{\partial X_2} + \eta _{13}\frac{\partial u_1}{\partial X_3} \Bigl ) \end{aligned}$$
